# Validation of the Chinese EORTC chronic lymphocytic leukaemia module – application of classical test theory and item response theory

**DOI:** 10.1186/s12955-020-01341-z

**Published:** 2020-04-07

**Authors:** Dong Dong, Jun Jin, Simone Oerlemans, Siyue Yu, Shenmiao Yang, Jianfeng Zhu, Richard Huan Xu

**Affiliations:** 1 JC School of Public Health & Primary Care, The Chinese University of Hong Kong, Hong Kong, SAR China; 2 Department of Sociology, School of Social Sciences, Tsinghua University, Beijing, China; 3 Netherlands Comprehensive Cancer Organization, Eindhoven, Netherlands; 4 Peking University Institute of Hematology, Peking University People’s Hospital, Beijing, China; 5 School of Social Development and Public Policy, Fudan University, Shanghai, China

**Keywords:** Health-related quality of life (HRQoL), Chronic lymphocytic leukaemia, EORTC-CLL17, China, Psychometrics, Item response theory

## Abstract

**Purpose:**

The association of chronic lymphocytic leukemia (CLL) with health-related quality of life (HRQoL) is rarely studied globally. This study evaluated the psychometric properties of the EORTC-Chronic Lymphocytic Leukaemia (CLL17 [phase III]) module, a newly developed assessment on CLL patients’ HRQoL, among Chinese CLL patients.

**Methods:**

The Chinese CLL17, comprised of three subscales (symptom burden [SB], physical condition [PC] and worries/fears [WF]), was provided by the developer team through EORTC. A cross-sectional online survey was conducted to collect data. The classical traditional theory (CTT) and the item response theory (IRT) were used to evaluate the psychometric properties of CLL17. Internal consistency reliability was determined by the Cronbach’s alpha and item-total correlation. Dimensionality was verified through confirmatory factor analysis (CFA). Convergent validity was also assessed. The generalized partial credit model was used for the IRT. The difficulty, discrimination, item fit, and differential item functioning (DIF) were calculated to assess the instrument’s psychometric properties.

**Results:**

In all, 318 patients, aged between 26 and 82 years, completed the questionnaire. A good level of internal reliability was achieved (Cronbach’s alpha = 0.92). The item-total correlation coefficient ranged from 0.46 to 0.72. There was a mid-to-high correlation between CLL17 and domains of EQ-5D and QLQ-C30. The IRT model showed a satisfactory homogeneity, item fit and good discrimination of items, except for item 4, 6 and 16 (< 1.0). low information provided by item 16 and 17. SB and PC provided more information with theta > 0, whereas WF provided more information with theta < 0. Item 17 perform inconsistently for respondents from different age groups (DIF).

**Conclusion:**

The EORTC-CLL17 Chinese version shows acceptable reliability and validity, making it a valuable instrument to evaluate the impact on the HRQoL of Chinese CLL patients.

## Introduction

Chronic lymphocytic leukemia (CLL) is the most common leukemia among adults, affecting the white blood cells that develop slowly over time [1]. In the United States, the National Cancer Institute reported that the number of new cases of CLL was 4.9 per 100,000 that annually caused 1.2 per 100,000 deaths [2]. Cancer Research United Kingdom stated that between 2014 and 2016, nearly 1% of the total cancer cases reported in the UK included people living with CLL [3]. In Asian countries, including China, although the CLL was less common than western countries, the prevalence is increasing dramatically in recent years. According to the latest research reported by China’s National Cancer Centre, lymphoma was found to be one of the top ten causes of cancer death in cities, and one of the top ten most common cancers among men. CLL, as one of the most common lymphomas, is estimated to affect an increasing number of the Chinese population [4]. Since the incidence rate of CLL rises with age, and aging is becoming an increasingly significant problem in China, the morbidity and mortality of CLL are expected to rise rapidly in the next few decades [5]. Hence, even though the prevalence of CLL is not “high” for now, it still deserves attention and research from academia.

In the last two decades, the treatment of CLL has dramatically improved and significantly extended patients’ life expectancy [1, 6]. The National Institute of Health reported that from 2009 to 2015, the five-year survival rate of CLL was 85.1%, which is higher than most other cancers for both males and females in the U.S. [2]. Compared with the flourishing studies on treatments of CLL disorders, research assessing the health-related quality of life (HRQoL) of CLL patients is scarce. Although some have encouraged the adoption of cancer-specific measures, such as EORTC QLQ-C30 [7], to assess CLL patients’ HRQoL in previous studies, others have argued that these may be unsuitable for measuring the effectiveness of interventions or policies focusing on CLL care due to the bias and uncertainties brought up by non-CLL specific measures [8]. Recently, the application of FACT-Lymphoma (FACT-L), an instrument developed to assess the HRQoL of patients living with all types of lymphoma, has increased [9–11]. However, lymphoma has many different subtypes, such as Hodgkin’s Lymphoma, Non-Hodgkin’s Lymphoma, and so on. Each subtype is unique with its own causes, symptoms, and methods of treatment, and thus its impact on patients’ HRQoL may also differ. Whereas the FACT-L can assess the overall HRQoL of lymphoma patients, the nuances in differences among patients with different subtypes may not be detected. It is, therefore, imperative to develop a set of scales to assess a disease-specific HRQoL among patients with different subtypes of lymphoma.

Recently, a growing number of targeted therapies (e.g., ibrutinib, idelalisib, venetoclax, obinutuzumab), used as a monotherapy or in combination, have been introduced for CLL treatment. As the 2020 updated recommendations on CLL diagnosis and treatment suggest, there is an “impressive and historically unique chance” for the long-term control of the disease. To achieve this, CLL management is also critical besides clinical trials [12]. Accordingly, the assessment of CLL patients’ HRQoL by using a disease-specific measure seems imperative. Moreover, it has also been argued that the use of general measures, such as the SF-12, may limit a full appraisal of outcome difference between arms in clinical trials [13]. With a standardized and validated HRQoL scale, the sensitivity to detect functional limitations and symptoms in future trials on CLL patients is expected to increase.

In 2018, the Quality of Life Group of the European Organisation for Research and Treatment of Cancer (EORTC) developed a series of lymphoma-specific measures to assess the HRQoL of patients with different types of lymphoma [13]. Among them, EORTC QLQ-CLL17 (CLL17), a CLL-specific scale, was introduced. Although the scale is in phase III of development, which means that it can be used for validation within different social and cultural contexts but should not be regarded as the final version of the scale, it has been reported to have good reliability (the Cronbach’s alpha ranges between 0.85 and 0.88) and validity when using a sample of 86 participants from five Western countries [13]. The new scale provides an advanced assessment of the HRQoL, including both the physical and mental dimensions of patients living with CLL. In China, although the population of patients affected by CLL has expanded in recent years [5], studies on the impact of CLL on patient’s HRQoL are still nascent. It is important to provide a valid, reliable, and responsive measure to evaluate the impact of CLL and associated interventions on the HRQoL changes. Therefore, this study evaluated the psychometric properties of CLL17 among Chinese CLL patients using the methods based on both classical traditional theory (CTT) and item response theory (IRT).

## Method

### Study design and participants

A cross-sectional study was conducted to survey the medical, social, and economic status of people affected by rare diseases in China, which was approved by the Chinese University of Hong Kong Institutional Review Board committee (Ref No.: SBRE-18-268). In all, 4225 patients with lymphoma participated in the survey and completed the questionnaire. Of these, data from 318 patients who reported having CLL were used in this study. The inclusion criteria were patients 1) who were ≥ 18 years old, 2) with a confirmed clinical diagnosis of CLL, 3) who could complete the questionnaire independently, and 4) were native Chinese speakers. Patient with cognitive problems were excluded from the survey. Respondents belonged to 30 of the total 34 provinces of China and were aged between 26 and 82 years. All the CLL patients were recruited via *House086*, the largest national lymphoma patient organization in China, between May and July 2019. *House086*, established in 2011, aims to help patients with all types of lymphomas in China and currently has more than 40,000 registered members (lymphoma patients and their families only). The survey was conducted online. All participants were asked to read the consent form presented on the first page of the online survey. If they agreed to participate, they clicked “next page” and were directed to the main body of the questionnaire.

The questionnaire comprised seven parts, which included demographics, HRQoL, social support, and others. Respondents resided in 30 provinces across China, and the three largest groups were from *Jiangsu* (29 respondents), *Henan* (24 respondents), and *Zhejiang* (23 respondents). All responses were recorded anonymously and treated with strict confidentiality. The sample size was calculated based on the assumption of conducting psychometric evaluation [14, 15]. Considering that CLL is listed as a rare disease in China, a minimum sample of 200 was needed.

### The instrument - EORTC CLL17 Chinese version

The CLL17 module is a supplementary questionnaire module to be employed in conjunction with the EORTC-C30 to evaluate the HRQoL of patients living with CLL. The CLL17 comprises 17 items divided into three subscales: ‘Symptom burden’ (SB, 6 items, 1–6), ‘Physical condition/fatigue’ (PC, 4 items, 7–10), and ‘Worries/fears about the health and functioning’ (WF, 5/7 items, 11–17, the last two items are required only when the patients’ conditions are applicable). The questions are answered using a 4-point response scale (1 = Not at all, 2 = A little, 3 = Quite a bit, 4 = Very much) to indicate the extent of the problem experienced by the respondents for each item during the past week(s) [13]. The scores of each subscale are calculated based on the EORTC’s protocol and then transformed to a 0–100 scale. The first step is calculating a raw score for each subscale (Raw score = {(*I*_1_ + *I*_2_+ … *I*_n_)/n}, *I*_*1*_ is the item 1, n is the number of the item in that subscale). The second step is linear transformation to obtain the standardized score S (0–100, S = {Raw score − 1}/range} *100). The simplified Chinese version of CLL17 (CLL17-C) was provided by the developer team through EORTC. It was translated by a professional translation service provider, specializing in the translation of patient-reported outcome measures. They followed the standard EORTC translation process as described in the EORTC’s Translation Manual (The Manual is available under request). Translators were selected following ISO requirements.

### Data analysis

In this study, data were analyzed based on the methods from both CTT and IRT using R (R foundation, Austria). The *p*-value was set at ≤0.05. The demographic data are reported as percentages, means, standard deviations (*SD*), and ranges. The feasibility of the questionnaire was evaluated based on the calculation of the percentage of missing data and the time to complete the questionnaire. The ceiling and floor effects, which reflect the majority of the values occurring at the upper or bottom limit of the scale, were calculated (< 15% is acceptable [16]).

For the CTT methods, the internal consistency reliability was reported, which included 1) Cronbach’s alpha coefficient (α), α > 0.7 was considered as acceptable [14], and 2) the item-total correlation coefficient, wherein an item with a low corrected item-total correlation (*r* < 0.3) was considered to be removed from the scale [17]. Confirmatory factor analysis (CFA) was used to evaluate the dimensionality of CLL-C. The comparative fit index (> 0.90), Tucker-Lewis index (> 0.90), standardized root mean square residual (< 0.08), and root mean square error of approximation (< 0.08) [14] were used to evaluate the performance of the scale. The EuroQol five-dimension five-level (EQ-5D-5 L) and the EORTC QLQ-C30 were used to evaluate the convergent validity of CLL-C. The EQ-5D is the most commonly used generic instrument to describe and value health worldwide. It has five dimensions (Mobility, Self-care, Usual activities, Pain/Discomfort, and Anxiety/depression), and each dimension has three (3 L) or five (5 L) levels. The combinations of the dimensions and levels could generate a single summary index score for health status used in the economic evaluation of health care interventions [18]. The EORTC QLQ-C30 is a questionnaire developed to specifically assess the quality of life of cancer patients [19]. It has five functional scales (Physical, Role, Cognitive, Emotional, and Social Functioning), three symptom scales (Fatigue, Pain, and Nausea/Vomiting), a Global Health Status scale, and six single items (Constipation, Diarrhea, Insomnia, et al.). Scores for each scale range from 0 to 100. For functional scales and Global Health Status, a higher score indicates better functioning or HRQoL. However, for symptom scales and single items, a higher score indicates worse status.

For the IRT evaluation, a multidimensional extension of the generalized partial credit model (GPCM) was applied [20], which means the discrimination of all the items were estimated separately. The key assumptions, i.e., monotonicity (item-category characteristic curves, ICCs) and local dependence (discrimination< 4), were confirmed with the analysis. The indicators of difficulty (b), discrimination (a), and item fit (the *p*-value of S-χ^2^ < 0.05) were calculated for each item, along with the item information curves (IICs) and test information curve (TIC) [21]. Furthermore, differential item functioning (DIF) was also checked for possible item bias caused by responses from different subgroups (sex and age group) in the sample, followed by the Monte Carlo simulated empirical criteria [17]. The detailed information of DIF detection can be found in Choi et al. [22].

## Results

### Demographics

Table 1 presents the demographics of the respondents. In total, 318 respondents completed the questionnaire with a mean age of 55 years (26~82 years). About 61.9% were male, 45.6% received a tertiary or above educational certificate, and 40% were retired. Moreover, the average duration of the lymphoma was 3.91 years (*SD* = 3.05).

**Table 1:** Demographics of respondents

	Number	Percent
Sex		
Male	197	61.9
Female	121	38.1
Age (mean, range [sd])	55.02 (26 ~ 82)	10.47
Education		
No/Primary	35	11.0
Secondary	138	43.4
Tertiary or above	145	45.6
Employment		
Full-time employed	95	29.9
Part-time employed	6	1.9
Farming	27	8.5
Unemployed	55	17.3
Retired	126	39.6
Housewife	9	2.8
Family annual income (mean, range [sd])	199,266 (2000~15,000,000)	10,246
Years with disease (mean, range [sd])	3.91 (1~15)	3.05
Number of children (mean, range [sd])	2.39 (1~4)	0.66

*sd* Standard deviation, *min* minimum, *max* maximum

### Feasibility

The percentage of the missing data at the item level was zero. Given that the CLL-C was embedded in a survey to investigate the health conditions of the lymphocytic patients, estimating the exact time required to complete the CLL-C was impossible. However, based on our pilot study, and the responses from some participants, the time to complete the CLL-C was less than 5 min, which is entirely acceptable.

### Item statistics and reliability

Table 2 shows the item statistics and internal consistency reliability of CLL-C. Item 15 (WF subscale) had the highest average score (2.94), whereas item 7 (PC subscale) had the lowest average score (1.66). There were, overall, 10 items with an average score greater than 2 points [1–4]. The Cronbach’s alpha for the overall was 0.92. For SB, PC, and WF, α was 0.81, 0.85, and 0.91, respectively. Additionally, 12/17 items had α ≥ 0.85. The value of the item-total correlation coefficient ranged between 0.46 and 0.72, and all reached the predefined criteria. No item violated the assumption that α would be smaller without a particular item. The ceiling effect for items ranged from 1.88 to 34.27% (6/17 presented ceiling effect), whereas the floor effect ranged from 5.35 to 41.82% (12/17 presented floor effect).

**Table 2:** Item statistics and internal consistency reliability of QLQ-CLL17

	N	Range	Mean	sd	%Ceiling	%Floor	Internal consistency reliability		
							Cronbach’s alpha	Alpha if item deleted	Item-total correlation
							0.92		
SB	318	0–100	30.01	17.85			0.81		
CLL1	318	1–4	1.68	0.63	2.51	41.82	0.78	0.56	0.62
CLL2	318	1–4	2.00	0.72	3.45	22.01	0.77	0.63	0.74
CLL3	318	1–4	1.82	0.73	2.52	34.28	0.76	0.64	0.75
CLL4	318	1–4	1.78	0.69	2.52	34.91	0.80	0.48	0.52
CLL5	318	1–4	2.22	0.81	5.97	17.92	0.76	0.63	0.70
CLL6	318	1–4	1.88	0.84	6.28	35.53	0.80	0.49	0.53
PC	318	0–100	30.45	19.82			0.85		
CLL7	318	1–4	1.66	0.70	2.20	45.28	0.85	0.59	0.64
CLL8	318	1–4	2.15	0.71	5.35	13.21	0.86	0.74	0.80
CLL9	318	1–4	1.88	0.71	1.88	29.56	0.82	0.68	0.74
CLL10	318	1–4	1.95	0.75	4.09	26.10	0.87	0.74	0.81
WF	318	0–100	56.66	26.74			0.91		
CLL11	318	1–4	2.75	0.94	27.98	6.92	0.88	0.82	0.86
CLL12	318	1–4	2.88	0.95	34.27	5.97	0.89	0.78	0.83
CLL13	318	1–4	2.78	0.94	28.94	6.29	0.89	0.81	0.85
CLL14	318	1–4	2.64	1.07	30.18	15.41	0.89	0.75	0.80
CLL15	318	1–4	2.94	0.94	35.53	5.35	0.89	0.81	0.85
CLL16	239	1–4	2.36	0.95	15.48	17.99	0.92	0.51	0.56
CLL17	243	1–4	2.56	1.05	25.51	17.28	0.91	0.63	0.68

*sd* Standard deviation, *SB* symptom burden, *PC* physical condition/fatigue, *WF* Worries/fears about health and functioning

### Construct validity

The result of CFA supports the three-subscale construct of CLL-C. However, the model of 15 items (without items 16 and 17) showed a better performance than the model of 17 items (Table 3). Table 4 presents the results of known-group validity. We identified that patients receiving treatment were prone to report a higher score (low HRQoL) on all three subscales. The scores of SB and PC correlated significantly with treatment for CLL patients.

**Table 3:** Results of confirmatory factor analysis (two models)

Model (three subscales)	Chi-square	df	CFI	TLI	RMSEA	SRMR
EORTC-CLL17 with item 16 and item 17	361.39	116	0.90	0.871	0.090	0.073
EORTC-CLL17 without item 16 and item 17	268.97	87	0.936	0.923	0.080	0.054

*df* degree of freedom, *CFI* Comparative fit index (> 0.9), *TLI* Tucker-Lewis index (> 0.9), *RMSEA* root mean square error of approximation (< 0.08), *SRMR* standardized root mean square residual (< 0.08)

**Table 4:** Known-groups validity between patients receiving treatment and not receiving treatment

	Treatment (*n* = 228)		No treatment (*n* = 49)		Difference (95% C.I.)	*p*-value
	Mean	sd	Mean	sd		
SB (item 1–6)	31.75	17.41	20.41	14.98	11.34 (6.5 ~ 16.17)	< 0.001
PC (item 7–10)	31.91	20.02	24.32	15.2	7.59 (2.53 ~ 12.64)	0.003
WF (item 11–17)	57.56	27.14	52.84	24.97	4.72 (−3.23~12.68)	0.24

There are four kinds of treatment: chemotherapy, radiation therapy, immunotherapy, or surgery

95% C.I., 95% confidence interval

*p*-value was calculated based on the robust one-way ANOVA on trimmed means suggested by Wilcox

*SB* symptom burden, *PC* physical condition/fatigue, *WF* Worries/fears about health and functioning

### Convergent validity

Compared with EQ-5D-5 L, there was a strong correlation between WF and anxiety/depression of EQ-5D-5 L (*r* = 0.573), followed by the correlation between SB and pain/discomfort (*r* = 0.473). Compared with QLQ-C30, the PF of CLL-C correlated strongly with fatigue (*r* = 0.713), and SB and PF of CLL-C seem to have a closer relationship with domains of QLQ-C30 than the WF. All the correlation coefficients were statistically significant (Table 5).

**Table 5:** Convergent and discriminant validity of QLQ-CLL17 (correlation coefficient)

	WF	SB	PC
EQ-5D-5 L			
Mobility	0.242***	0.435***	0.413***
Self-care	0.202***	0.292***	0.314***
Usual activities	0.268***	0.410***	0.397***
Pain/discomfort	0.298***	0.473***	0.412***
vAnxiety/depression	0.573***	0.440***	0.439***
Utility	−0.457***	− 0.569***	− 0.549***
QLQ-C30^a^			
Global health status	−0.446***	− 0.537***	− 0.577***
Functional scales			
Physical functioning	−0.405***	−0.633***	− 0.655***
Role functioning	−0.41***	− 0.55***	− 0.563***
Emotional functioning	− 0.634***	− 0.583***	− 0.568***
Cognitive functioning	− 0.511***	− 0.614***	− 0.601***
Social functioning	− 0.638***	−0.53***	− 0.564***
Symptom scales			
Fatigue	0.475***	0.679***	0.713***
Nausea and vomiting	0.243***	0.444***	0.414***
Pain	0.397***	0.65***	0.538***
Dyspnoea	0.424***	0.645***	0.652***
Insomnia	0.437***	0.488***	0.491***
Appetite loss	0.393***	0.536***	0.524***
Constipation	0.204***	0.38***	0.367***
Diarrhea	0.164***	0.285***	0.243***
Financial difficulties	0.55***	0.461***	0.419***
QLQ-CLL17			
WF	1.00	0.477***	0.544***
SB		1.00	0.734***
PC			1.00

*SB* symptom burden, *PC* physical condition/fatigue, *WF* Worries/fears about health and functioning

^a^The score or global health status and functional scales of QLQ-C30 and utility of EQ-5D-5 L were reversed: a higher score means a better status and a lower score means a worse status

* *p*< 0.05; ** *p*< 0.01; *** *p*< 0.001

### IRT analysis - model fit

Table 6 indicates the discrimination and difficulty parameters for CLL-C. The discrimination of 17 items ranged between 0.761 and 3.877. The value of items 2, 3, 8, 10, 11, 12, 13, and 15 fell within the range greater than 3.0, which indicates that these items can distinguish individuals with either lower or higher HRQoL, corresponding with the latent trait sensitively. The *p*-value of S-χ^2^ indicates that all the items fit the scale, except for item 7 (*p*-value = 0.05), which might have a minor misfit.

**Table 6:** Discrimination and difficulty parameters for QLQ-CLL17

	Discrimination	Threshold indices			S-χ^2^	*P* value
	a	b1	b2	b3		
SB						
CLL1	1.265	−0.286	2.389	2.214	13.52	0.330
CLL2	3.112	−0.860	0.992	2.062	17.02	0.074
CLL3	3.166	−0.440	1.203	2.266	11.65	0.309
CLL4	0.880	−0.692	2.634	2.515	9.64	0.885
CLL5	1.279	−1.317	0.634	2.319	19.86	0.281
CLL6	0.761	−0.579	2.216	1.697	19.76	0.409
PC						
CLL7	1.515	−0.290	1.979	2.340	16.52	0.050
CLL8	3.655	−1.226	0.797	1.727	6.16	0.291
CLL9	2.603	−0.624	1.174	2.465	3.77	0.438
CLL10	3.531	−0.699	1.053	1.866	2.18	0.704
WF						
CLL11	3.702	−1.58	−0.121	0.557	10.53	0.65
CLL12	3.624	−1.667	−0.266	0.354	19.68	0.103
CLL13	3.568	−1.647	−0.152	0.526	12.62	0.478
CLL14	2.573	−1.143	0.098	0.395	10.86	0.900
CLL15	3.877	−1.707	−0.374	0.329	12.88	0.458
CLL16	0.776	−1.413	1.043	1.268	19.51	0.882
CLL17	1.022	−1.150	0.473	0.531	28.59	0.330

*SB* symptom burden, *PC* physical condition/fatigue, *WF* Worries/fears about health and functioning, a = discrimination, b = difficulty

### IRT analysis - item-category characteristic curves

**Fig. 1:**
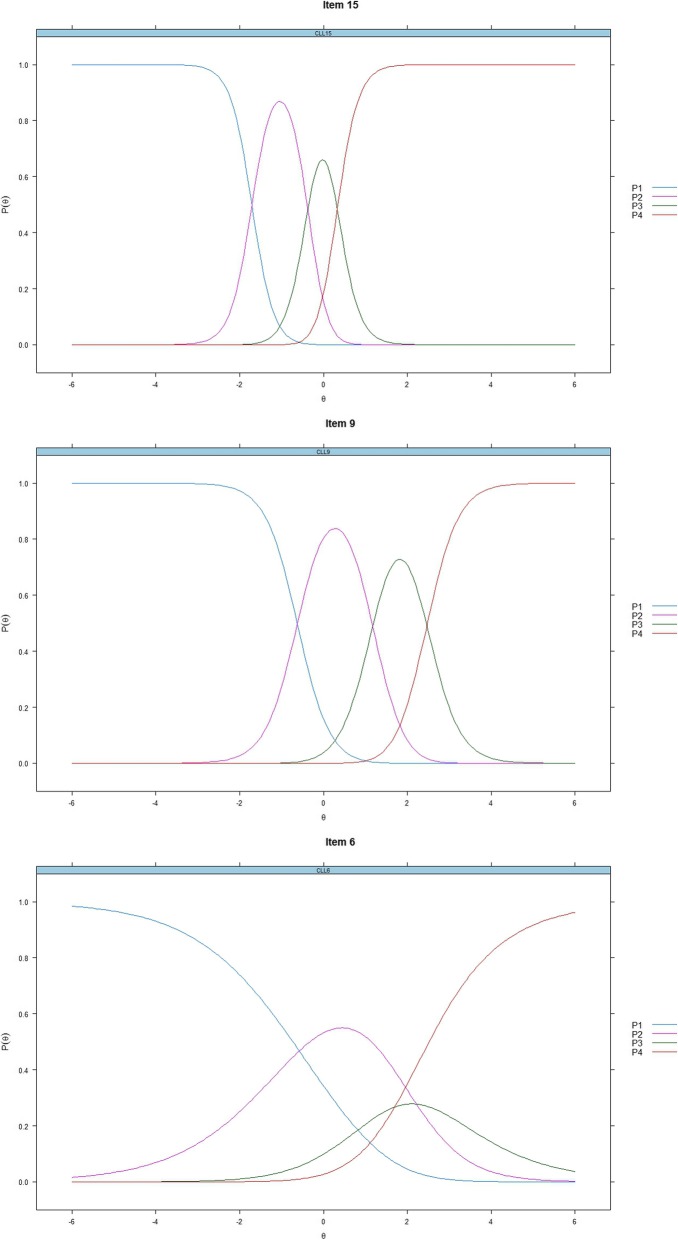
Item-category characteristic curves – Item 6, 9 and 15

Figure 1 graphically presents the item-category characteristic curves of items 6, 9, and 15, showing how the items relate to each latent trait. The selected three items reflected the different (high, moderate, and low) levels of discrimination of all items. Item 15 had the highest discrimination (a = 3.877), item 9 was moderate (a = 2.603), and item 6 had the lowest discrimination (a = 0.761). Although the order of categories’ thresholds for most items (e.g., item 9 and item 15) was good (the interaction of ‘harder’ options had a higher theta value than the interaction of ‘easier’ options), the thresholds for very few items (e.g., item 6) had an overlap among the response categories, which led to those categories offering little in terms of placing a respondent on the scale. For the location parameter, the response category for item 15 was endorsed at lower levels of the latent trait (WF), but items 9 and 6 were endorsed at higher levels (PC and SB). All the Item-category characteristic curves are shown in the supplementary file.

### IRT analysis - information function curves

**Fig. 2:**
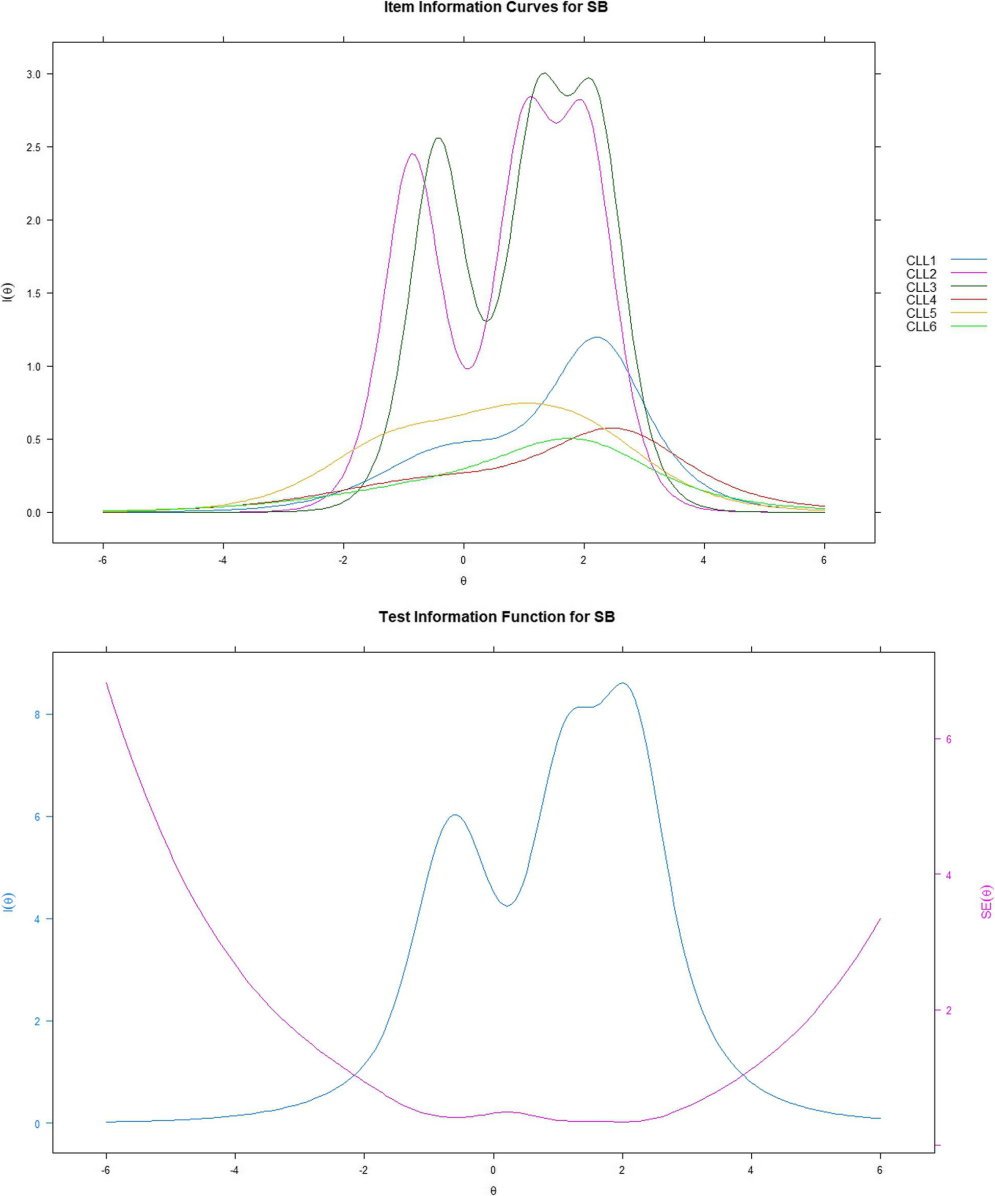
Item and test information function for SB; SB = symptom burden; Latent trait (Theta) is shown on the horizontal axis, and the amount of information is shown on the vertical axis

**Fig. 3:**
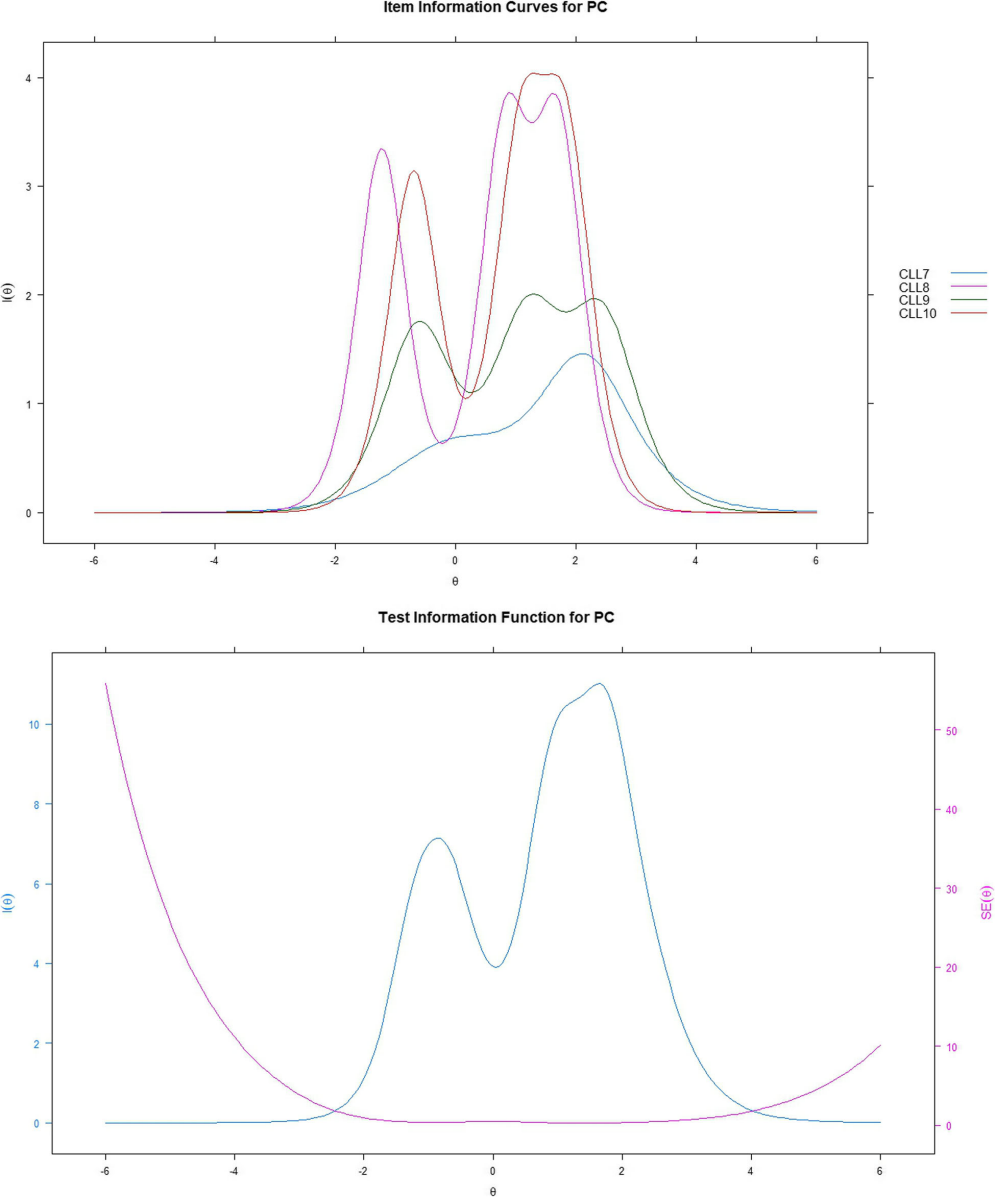
Item and test information function for PC; PC = physical condition/fatigue; Latent trait (Theta) is shown on the horizontal axis, and the amount of information is shown on the vertical axis

**Fig. 4:**
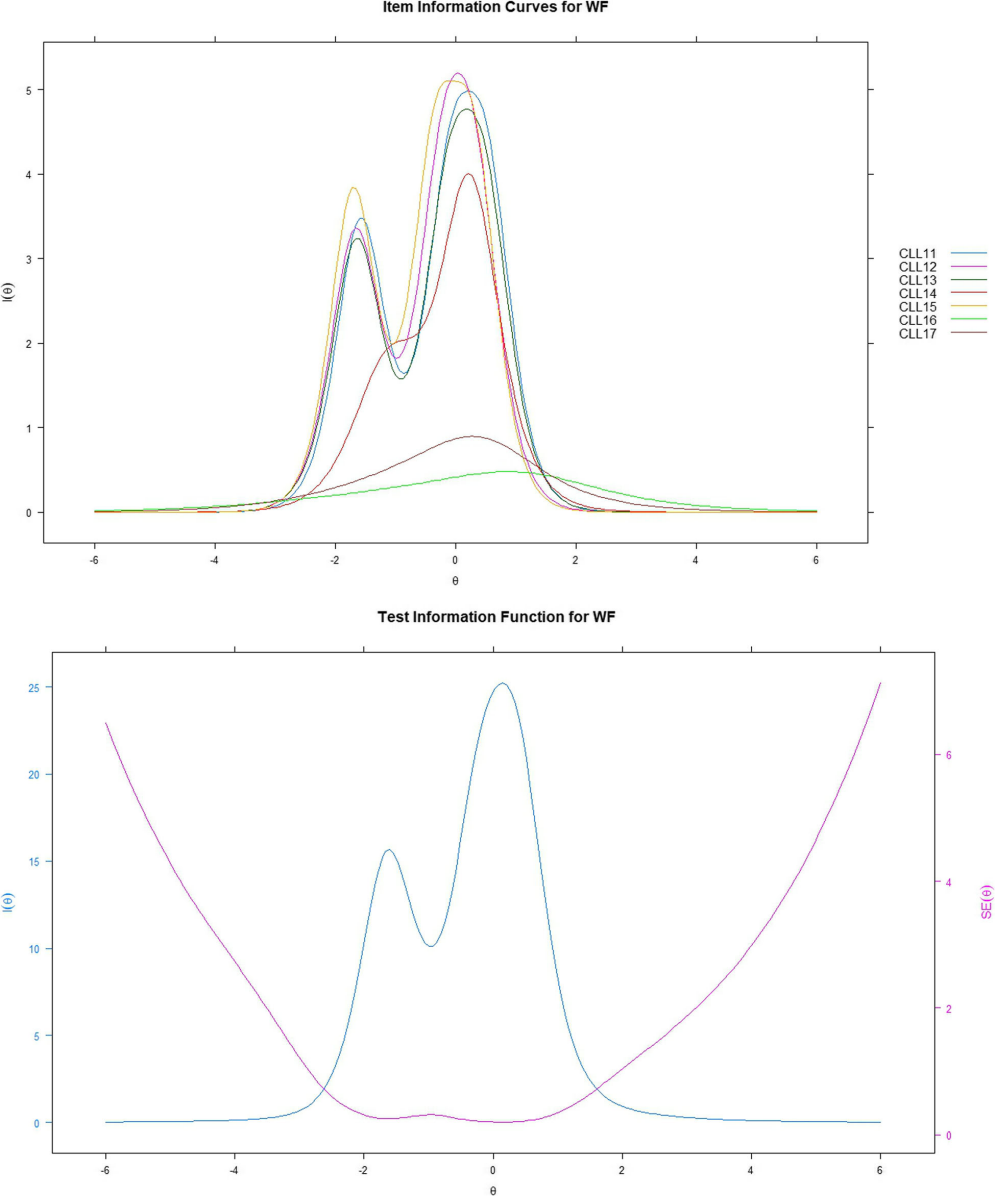
Item and test information function for WF; WF=Worries/fears about health and functioning; Latent trait (Theta) is shown on the horizontal axis, and the amount of information is shown on the vertical axis

Figures 2, 3, 4 show item and test information curves for three subscales separately. Information function curves demonstrate the precision and information provided by each item [23]. These curves graphically present the information functions of all the items on a scale, demonstrating the precision of a scale across different levels of the targeted latent trait [15]. Items 2 and 3 in the SB subscale, and items 8 and 10 in the PC subscale provided more information than the other items. For WF, however, items 16 and 17 seem to provide the least information. The test information curves were sufficiently informative for the middle level of the latent trait with the range of theta from 1 to 2.5, 1.5 to 2.5, and − 0.5 to 0.5 for SB, PC, and WF, respectively. For SB and PC, the overall distribution of information revealed that the subscales provided more information alongside the trait region associated with theta ranges between zero and six; however, for WF, more than half of the information was provided alongside the trait region associated with theta ranges between − 6 and zero.

### DIF analysis

Item 17 showed a uniform DIF when considering the respondents from different age groups. It revealed that the concerns about continuing to work or study improve with increased age for CLL patients even when they report the same levels of WF. However, when looking at differences in pseudo-*R*^2^ values, the magnitude of *R*^2^ is smaller than 0.13, suggesting that the effect size of DIF is very negligible [24]. The figure of DIF is presented in the supplementary file.

## Discussion

CLL-C is a new and valid instrument developed by EORTC to assess the HRQoL in patients with CLL [13]. Our study used its simplified Chinese version and evaluated its psychometric properties. Our results indicate that the reported mean scores of SB and PC were similar to the mean scores reported by patients using the original English version [13]. For the WF (emotional sub-scale), however, the mean score was much higher than the scores reported in the original study for both with and without items 16 and 17. Previous studies identified CLL as one of the most common forms of leukemia in Western countries, accounting for 25% of all leukemia in adults. However, in Asian countries, including China, CLL is rare [25]. Regarded as a rare disease in China, patients diagnosed with CLL are prone to experience more emotional burden or depression than their Western counterparts. Discussions about such relationships may go beyond the purpose of this study. Therefore, further investigations are encouraged.

Our psychometric evaluation demonstrates that the multi-trait scaling structure of CLL17-C is generally similar to that of its original English version [13]. The CLL17-C has good internal consistency reliability, as supported by both Cronbach’s alpha and item-total correlation coefficient. Moreover, statistically significant correlations between the subscales of CLL17-C, the EQ-5D-5 L, and QLQ-C30 suggest that the subscales of CLL17-C could assess the distinct components of the construct of HRQoL. For the known-group validity, the results also meet our expectations that the patients who received clinical interventions got higher scores on CLL17-C (worse status). The findings suggest that CLL17-C is capable of distinguishing patients based on the severity of their health status.

Based on the results of the IRT analysis, using the polytomous extension of the S-χ2 analysis, none of the items of CLL17-C were identified as a misfit at *p*-value < 0.05 after controlling the type I error rates [26]. The slope estimates of the 17 items ranged from 0.76 to 3.877 (12/17 greater than 1.5), indicating a modest variation in item discrimination. As expected, the higher categories have higher item locations, indicating the endorsement of severe HRQoL problems. For SB, b1 ranged between − 1.317 and − 0.286, b2 ranged between 0.634 and 2.634, and b3 ranged between 1.697 and 2.515. For PC, b1 ranged between − 1.226 and − 0.290, b2 ranged between 0.797 and 1.979, and b3 ranged between 0.727 and 2.340. For WF, b1 ranged between − 1.707 and − 1.143, b2 ranged between − 0.374, and 1.043 and b3 ranged between 0.329 and 1.268. However, WF items were much ‘easier’ to answer than the items of SB or PC. In other words, the CLL patients in our sample experienced emotional burdens more than physical discomfort. Moreover, although the discrepancy was not large, the thresholds of categories between b2 and b3 for items 1, 4, and 6 (SB sub-scale) were disordered. This might suggest that the response category “quite a bit” did not work consistently as intended in SB. However, considering our limited sample size, the different mechanism of parameter estimation using different IRT models [27], and no similar comparisons could be found around the world. Currently, it would be incorrect to conclude that the SB subscale of CLL17-C is not psychometrically sound. Further revisions are needed through more explorations.

The item information provided by SB, PC, and WF ranged from 2.24 to 9.49, 4.53 to 10.96, and 2.28 to 11.64, respectively. Based on Nunnally’s suggestion that values of information from 3.3 to 10 correspond to the Cronbach’s alpha ranges from 0.70 to 0.90 [28]. Therefore, the majority of the items indicate acceptable reliability. Items 4 (information: i = 2.58, α = 0.62), 6 (i = 2.24, α = 0.56), 16 (i = 2.28, α = 0.57), and 17 (i = 3.05, α = 0.68) may have some room for improvement. The TIC indicate that SB and PC had relatively smaller standard errors (SEs) at the high endpoint of theta than at the low endpoint of theta. However, for WF, the SEs were larger at both endpoints of theta, which mightindicate that when assessing the CLL, the WF subscale does not have a very reliable performance for patients with either high or low scores on emotional burdens related to CLL.

In this study, it is noteworthy that SB and PC items show a heavy ceiling effect, while there is an existence of a floor effect in the WF items. Moreover, the last two items of WF (items 16 and 17), which are not compulsory items, show both moderate ceiling and floor effects. Previous studies indicate that both the ceiling and floor effects are common in the HRQoL data [29–32]. A series of studies using SF-6D report the floor effects in a wide variety of clinical settings, whereas a strong correlation between the ceiling effect [33, 34] and the EQ-5D data have been identified [29, 31]. The floor effect might weaken the subscales’ ability (SB and PC) to capture the gains when patients have moderate or severe physical conditions or suffering. The magnitude of change experienced by CLL patients might be underestimated when using these two subscales, and the bias caused by the underestimation could potentially affect the evaluation of the effectiveness and efficiency of such interventions or policies. Similarly, the ceiling effect of items potentially threatens the responsiveness of WF, and the impacts are exactly the opposite of the floor effect. Currently, no other publications have reported the floor or ceiling effect of CLL17 in other language versions, and more information should be provided.

Item invariance is an important component of a good scale. If an item performed differently in subgroups after controlling for ability, the item is considered to have DIF [15]. To measure item invariance across different sub-samples of the CLL17-C, we set the two variables, sex and age, as the anchor items for all three subscales. The results indicate that the items in the three subscales performed consistently on assessing the HRQoL in terms of sex. However, item 17 of WF demonstrated a minor DIF in terms of the age group (≤50, 51–60, ≥61), which indicates less discrimination (flatter slope) for respondents younger than 50 than respondents older than 61. In other words, when patients worry or have fears about their health and functioning, patients of different ages do not give consistent answers to item 17. Nguyen et al. indicated that the possible reason for detecting DIF was how the item was administered [15]. Given that the data for this study came from an online self-report survey, it is unclear whether the process of data collection created some bias compared with a traditional paper-based questionnaire. However, until now, no DIF analysis was reported based on CLL17, and no other information could be provided at this moment. We explain this finding with caution.

As discussed above, the performance of item 17 in the CLL17-C appeared controversial, which seemingly leads to some negative effects, especially using the IRT model, on the estimation of the HRQoL of CLL patients. First, although the CFA suggests that the structure of CLL17-C is generally similar to its original English version, the 15-item (without items 16 and 17) version performed better than the full 17-item version. Secondly, the discrimination of item 17 was small (a = 1.022), indicating that item 17 may not differentiate patients with either higher or lower concerns about their work or education raised by CLL. Thirdly, the IIC demonstrates that item 17 provides the least information amongst all the items of CLL17-C.

Moreover, despite its negligible magnitude, item 17 shows a DIF in terms of age. The patients with CLL were usually older than patients reportedly living with other lymphomas. In our sample, the average age was 55 years. Whereas the participants’ average age in the study on the development of the CLL original version was 69 years [13]. Other studies of CLL also reported a similar demographics of their study sample, for example, 64 years in a study conducted in HK and Singapore [25], and 65 years reported by another UK study [35]. Thus, item 17, evaluating the impact of work and education on the HRQoL, may not provide useful information for CLL patients. However, since the CLL17 scale is still under the development of phase III, its final stage of development should include an improved version.

This study has some limitations. First, CLL patients with severe physical or psychological conditions might be underrepresented in our study. The absence of their information could lead to some bias in our results. Second, there is no golden standard or magical numbers that can be proposed to conduct the IRT analysis. The minimum requirement to test the questionnaire with polytomous response format was suggested to be 250 [36]; however, larger sample sizes, such as ≥500, are recommended considering different test purposes and IRT model selection [37, 38]. Our potentially insufficient sample size may have brought some bias in examining the fit of the model and over- or under-estimations of the parameters. A larger sample will be approached in the future to obtain a more precise measurement of CLL17-C item characteristics. Third, a high proportion of our participants came from economically developed areas in China, which might have led to potential selection bias. Lastly, we could not approach the patients with low willingness to participate in the survey since the data came from a self-report online survey, possibly leading to some selection bias.

## Conclusion

Although the treatments of CLL are fast developing, their effects on patients’ HRQoL in long-term care are still ambiguous. Our findings expand the understanding of HRQoL among CLL patients using a specific patient self-report outcome measure. This study provided comprehensive insights on the psychometric properties of CLL17-C using CTT analysis supplemented with IRT methods in a sample of Chinese patients. Both approaches showed that the three subscales had acceptable reliability and validity. However, given the limited sample size and the potential selection bias, further assessment of the measurement’s precision is encouraged to maximize the application of CLL17 regionally and internationally.

## Supplementary information

**Additional file 1: Table S1.** Confirmatory Factor Loadings of EORTC-CLL17 with item 16 and item 17. **Table S2.** Confirmatory Factor Loadings of EORTC-CLL17 without item 16 and item 17. **Figure S1.** ICC for subscale of SB. **Figure S2.** ICC for subscale of PC. **Figure S3.** ICC for subscale of WF. **Figure S4.** Graphically display of item 17 of QLQ-CLL17 that shows DIF based on age.

## Data Availability

Note applicable

## Abbreviations

CLL: Chronic lymphocytic leukemia
HRQoL: Health-related quality of life
EORTC: the Quality of Life Group of the European Organisation for Research and Treatment of Cancer Quality of life Cancer-30
FACT-L: The Functional Assessment of Cancer Therapy – Lymphoma
HL: Hodgkin’s Lymphoma
NHL: Non-Hodgkin’s Lymphoma
SF-12: Short form 12-item
CTT: Classical traditional theory
IRT: Item response theory
EQ-5D: EuroQol five dimension
EORTC QLQ-C30: EORTC quality of life cancer 30 items
SB: Symptom burden
PC: Physical condition/fatigue
WF: Worries/fears about the health and functioning
CFA: Confirmatory factor analysis
ICCs: Item-category characteristic curves
IICs: Item information curves
TIC: Test information curve
DIF: Differential item functioning

## References

[1] Buzaglo J, Miller M, Zaleta A, Johnson J, Mcmanus S, Karten C (2017). Chronic lymphocytic leukemia patient reported outcomes and quality of life: findings from the cancer experience registry. Blood.

[2] Chronic lymphocytic leukemia - cancer stat facts [Internet]. Available from: https://seer.cancer.gov/statfacts/html/clyl.html. Accessed 15 Sept 2019.

[3] Chronic lymphocytic leukaemia incidence statistics [Internet]. Available from: https://www.cancerresearchuk.org/health-professional/cancer-statistics/statistics-by-cancer-type/leukaemia-cll/incidence. Accessed 15 Sept 2019.

[4] Zheng RS, Sun KX, Zhang SW, Zeng HM, Zou XN, Chen R, et al. Report of cancer epidemiology in China, 2015. Zhonghua Zhong Liu Za Zhi. 2019;41(1):19. 10.3760/cma.j.issn.0253-3766.2019.01.005 30678413

[5] Yang S, Gale RP, Shi H, Liu Y, Lai Y, Lu J (2018). Is there an epidemic of chronic lymphocytic leukaemia (CLL) in China?. Leuk Res.

[6] O’Brien S, Furman RR, Coutre S, Flinn IW, Burger JA, Blum K (2018). Single-agent ibrutinib in treatment-naïve and relapsed/refractory chronic lymphocytic leukemia: a 5-year experience. Blood..

[7] Eichhorst BF, Busch R, Stilgenbauer S, Stauch M, Bergmann MA, Ritgen M (2009). First-line therapy with fludarabine compared with chlorambucil does not result in a major benefit for elderly patients with advanced chronic lymphocytic leukemia. Blood.

[8] Holtzer-Goor K, Schaafsma M, Joosten P, Posthuma E, Wittebol S, Huijgens P (2015). Quality of life of patients with chronic lymphocytic leukaemia in the Netherlands: results of a longitudinal multicentre study. Qual Life Res.

[9] Tam CS, Waller EK, Jaeger U, Fleury I, Mcguirk J, Holte H (2019). Prolonged improvement in patient reported quality of life (QoL) following Tisagenlecleucel infusion in adult patients (pts) with relapsed/refractory (r/r) diffuse large B-cell lymphoma (DLBCL): 19-month follow-up (FU) of the Juliet study. Biol Blood Marrow Transplant.

[10] Hess G, Rule S, Jurczak W, Jerkeman M, Santucci Silva R, Rusconi C (2017). Health-related quality of life data from a phase 3, international, randomized, open-label, multicenter study in patients with previously treated mantle cell lymphoma treated with ibrutinib versus temsirolimus. Leuk Lymphoma.

[11] Agarwal R, Bacchetta R, Bertaina A, Chu J, Chen P, Saini G (2019). Regulatory type 1 T cell infusion in mismatched related or unrelated hematopoietic stem cell transplantation (HSCT) for hematologic malignancies. Biol Blood Marrow Transplant.

[12] Hallek M (2019). Chronic lymphocytic leukemia: 2020 update on diagnosis, risk stratification and treatment. Am J Hematol.

[13] de Poll-Franse L, Oerlemans S, Bredart A, Kyriakou C, Sztankay M, Pallua S (2018). International development of four EORTC disease-specific quality of life questionnaires for patients with Hodgkin lymphoma, high- and low-grade non-Hodgkin lymphoma and chronic lymphocytic leukaemia. Qual Life Res.

[14] Streiner DL (2015). Health measurement scales [electronic resource] : a practical guide to their development and use. 5th ed. Norman GR, Cairney J, ebrary I, editors.

[15] Nguyen TH, Han H-R, Kim MT, Chan KS (2014). An introduction to item response theory for patient-reported outcome measurement. Patient Patient-Centered Outcomes Res.

[16] McHorney C, Tarlov A (1995). Individual-patient monitoring in clinical practice: are available health status surveys adequate?. An Int J Qual Life Asp Treat Care Rehabil - Off J Int Soc Qual Life Res.

[17] Xu RH, Cheung AW, Wong EL (2018). Development and validation of an instrument to measure patient engagement in Hong Kong special administrative region, China. Patient Prefer Adherence.

[18] Wong ELY, Xu RH, Cheung AWL (2019). Health-related quality of life among patients with hypertension: population-based survey using EQ-5D-5L in Hong Kong SAR, China. BMJ Open.

[19] Aaronson NK, Ahmedzai S, Bergman B, Bullinger M, Cull A, Duez NJ (1993). The European Organization for Research and Treatment of Cancer QLQ-C30: a quality-of-life instrument for use in international clinical trials in oncology. J Natl Cancer Inst.

[20] Coulacoglou C (2017). Psychometrics and psychological assessment: principles and applications. Saklofske DH, editor.

[21] Valdez JPM, Chu SKW. Examining the psychometric validity of the five-item gratitude questionnaire: an item response theory approach. J Psychoeduc Assess. 2018. 10.1177/0734282918816542.

[22] Choi S, Gibbons L, Crane PK (2011). Lordif: an R package fo rDetecting differential item functioning using iterative hybrid ordinal logistic regression/item response theory and Monte Carlo simulations. J Stat Softw.

[23] Xia J, Tang Z, Wu P, Wang J, Yu J (2019). Use of item response theory to develop a shortened version of the EORTC QLQ-BR23 scales. Sci Rep.

[24] Mair P (2018). Modern psychometrics with R.

[25] Chan TS-Y, Lee Y-S, Del Giudice I, Marinelli M, Ilari C, Cafforio L (2017). Clinicopathological features and outcome of chronic lymphocytic leukaemia in Chinese patients. Oncotarget..

[26] Benjamini Y, Drai D, Elmer G, Kafkafi N, Golani I (2001). Controlling the false discovery rate in behavior genetics research. Behav Brain Res.

[27] Bjorner JB (2019). State of the psychometric methods: comments on the ISOQOL SIG psychometric papers. J Patient-Reported Outcomes.

[28] Nunnally JC (1978). Psychometric theory.

[29] Färkkilä N, Torvinen S, Roine R, Sintonen H, Hänninen J, Taari K (2014). Health-related quality of life among breast, prostate, and colorectal cancer patients with end-stage disease. An Int J Qual Life Asp Treat Care Rehabil - Off J Int Soc Qual Life Res.

[30] Rajmil L, Abad S, Sardon O, Morera G, Pérez-Yarza EG, Moreno A (2011). Reliability and validity of the Spanish version of the TAPQOL: a health-related quality of life (HRQOL) instrument for 1- to 5-year-old children. Int J Nurs Stud.

[31] Mccaffrey N, Currow D, Ratcliffe J (2016). Health-related quality of life measured using the EQ-5D-5L: South Australian population norms. Health Qual Life Outcomes.

[32] Awl C, Ely W, RH X (2017). Examining the health-related quality of life using EQ-5D-5L in patients with four kinds of chronic diseases from specialist outpatient clinics in Hong Kong SAR, China. Patient Prefer Adherence.

[33] Hornbrook Mark C, Wendel Christopher S, Coons Stephen J, Grant Marcia J, Herrinton Lisa J, Mohler M (2011). Complications among colorectal cancer survivors: SF-6D preference-weighted quality of life scores. Med Care.

[34] Yang Y, Wong M, Lam C, Wong C (2014). Improving the mapping of condition-specific health-related quality of life onto SF-6D score. An Int J Qual Life Asp Treat Care Rehabil - Off J Int Soc Qual Life Res.

[35] Catovsky D, Richards S, Matutes E, Oscier D, Dyer M, Fbezares R (2007). Assessment of fludarabine plus cyclophosphamide for patients with chronic lymphocytic leukaemia (the LRF CLL4 trial): a randomised controlled trial. Lancet..

[36] Robins RW, Fraley RC, Krueger RF (2007). Handbook of research methods in personality psychology.

[37] de Sá Junior AR, de Andrade AG, Andrade LH, Gorenstein C, Wang Y-P (2018). Response pattern of depressive symptoms among college students: what lies behind items of the Beck depression inventory-II?. J Affect Disord.

[38] Cappelleri JC, Jason Lundy J, Hays RD (2014). Overview of classical test theory and item response theory for the quantitative assessment of items in developing patient-reported outcomes measures. Clin Ther.

