# Supplementary material for: Validation of the Chinese EORTC chronic lymphocytic leukaemia module – application of classical test theory and item response theory
Source: Health Qual Life Outcomes. 2020 Apr 7;18:96. doi: 10.1186/s12955-020-01341-z (PMC7137502; doi:10.1186/s12955-020-01341-z)
Supplement: Supplementary file 1 — Additional file 1: Table S1. Confirmatory Factor Loadings of EORTC-CLL17 with item 16 and item 17. Table S2. Confirmatory Factor Loadings of EORTC-CLL17 without item 16 and item 17. Figure S1. ICC for subscale of SB. Figure S2. ICC for subscale of PC. Figure S3. ICC for subscale of WF. Figure S4. Graphically display of item 17 of QLQ-CLL17 that shows DIF based on age. [file 12955_2020_1341_MOESM1_ESM.docx]

Table S-1 Confirmatory Factor Loadings of EORTC-CLL17 with item 16 and item 17

|  | Estimate | Standard error |
| --- | --- | --- |
| SB |  |  |
| CLL1 | 0.305*** | 0.032 |
| CLL2 | 0.299*** | 0.032 |
| CLL3 | 0.274*** | 0.03 |
| CLL4 | 0.378*** | 0.037 |
| CLL5 | 0.279*** | 0.033 |
| CLL6 | 0.497*** | 0.051 |
| PC |  |  |
| CLL7 | 0.31*** | 0.032 |
| CLL8 | 0.155*** | 0.02 |
| CLL9 | 0.232*** | 0.026 |
| CLL10 | 0.177*** | 0.024 |
| WF |  |  |
| CLL11 | 0.229*** | 0.027 |
| CLL12 | 0.256*** | 0.028 |
| CLL13 | 0.244*** | 0.028 |
| CLL14 | 0.347*** | 0.039 |
| CLL15 | 0.209*** | 0.025 |
| CLL16 | 0.678*** | 0.065 |
| CLL17 | 0.693*** | 0.068 |

Table S-2 Confirmatory Factor Loadings of EORTC-CLL17 without item 16 and item 17

|  | Estimate | Standard error |
| --- | --- | --- |
| SB |  |  |
| CLL1 | 0.271*** | 0.024 |
| CLL2 | 0.292*** | 0.026 |
| CLL3 | 0.299*** | 0.027 |
| CLL4 | 0.344*** | 0.029 |
| CLL5 | 0.258*** | 0.027 |
| CLL6 | 0.48*** | 0.041 |
| PC |  |  |
| CLL7 | 0.269*** | 0.024 |
| CLL8 | 0.161*** | 0.017 |
| CLL9 | 0.22*** | 0.021 |
| CLL10 | 0.181*** | 0.019 |
| WF |  |  |
| CLL11 | 0.224*** | 0.022 |
| CLL12 | 0.224*** | 0.022 |
| CLL13 | 0.223*** | 0.022 |
| CLL14 | 0.342*** | 0.032 |
| CLL15 | 0.213*** | 0.021 |


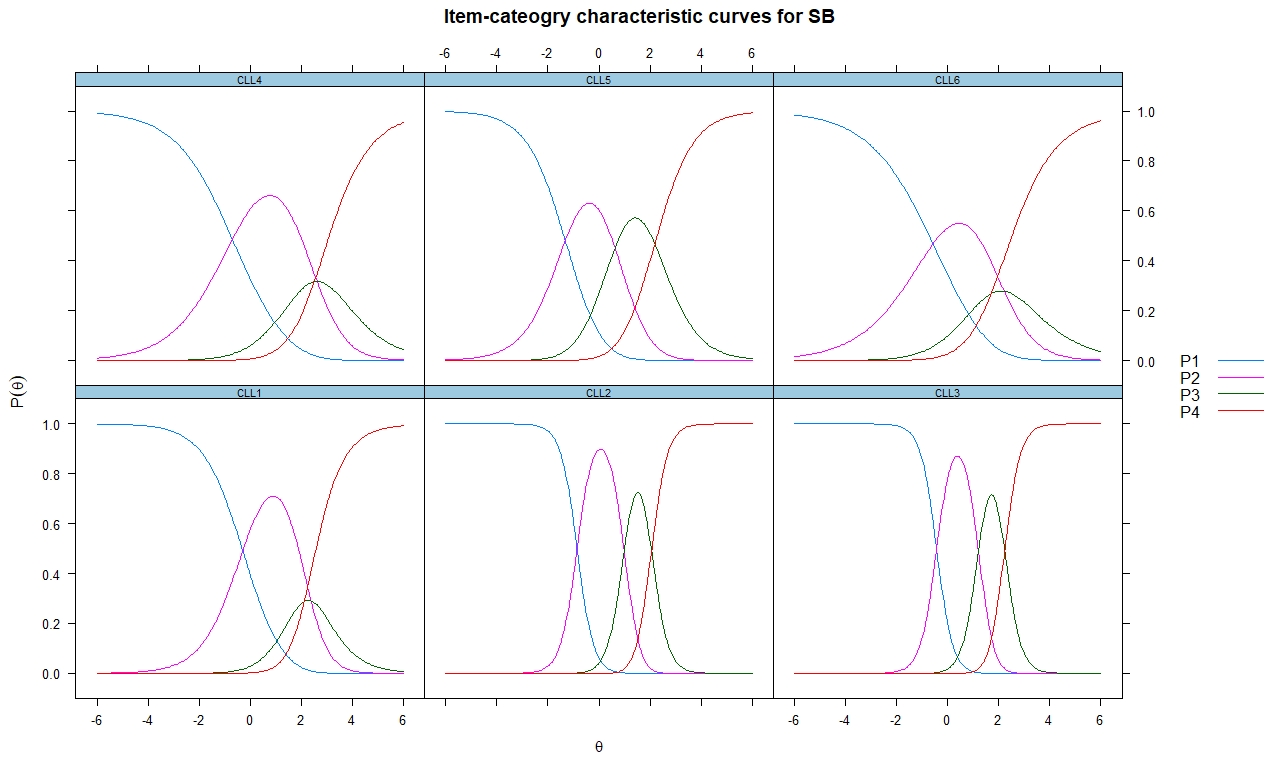


Fig S-1 ICC for subscale of SB


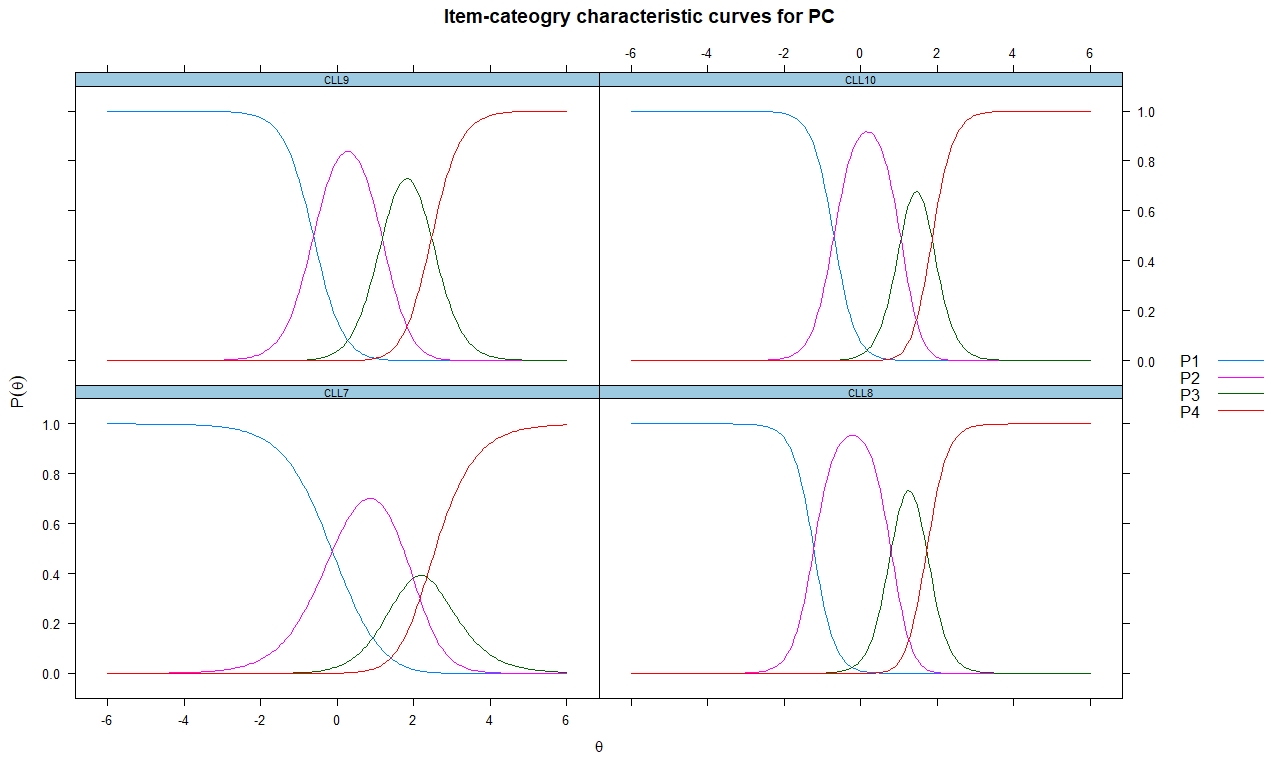


Fig S-2 ICC for subscale of PC


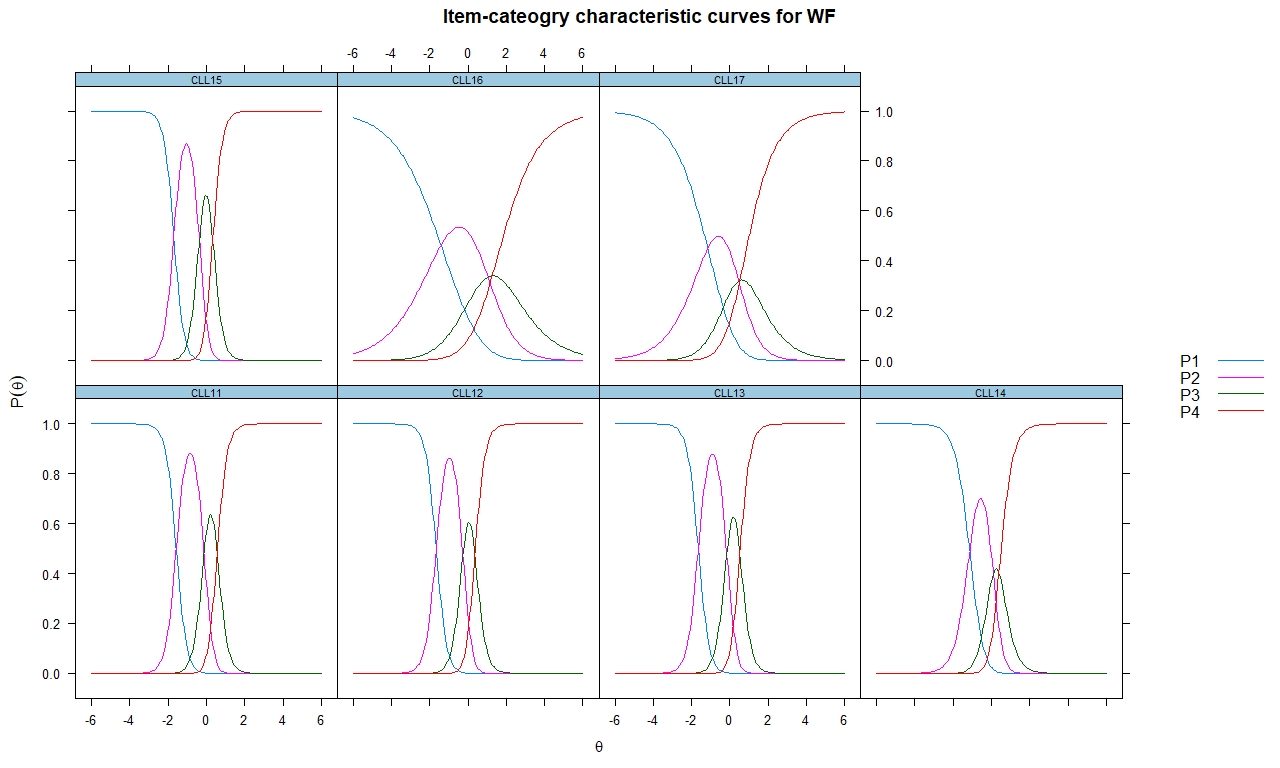


Fig S-3 ICC for subscale of WF


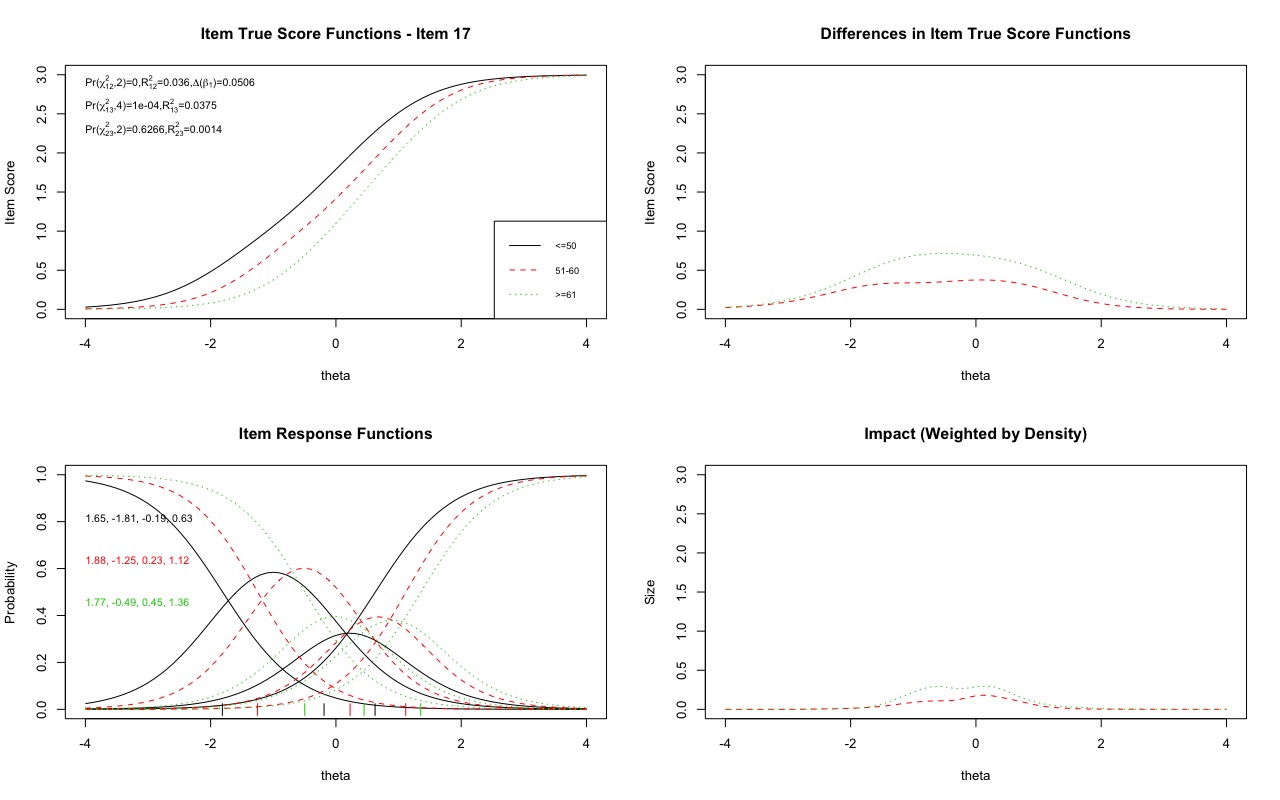


Fig S-4 Graphically display of item 17 of QLQ-CLL17 that shows DIF based on age

The introduction of the *House086*

House086 is a registered national patient organization. It has 10 full-time staff, all of whom are patients or family members of patients with lymphomas. House086 is also a member of the Lymphoma Coalition and the Union for International Cancer Control. All registered members of House086 must submit a copy of their diagnosis along with proof of their identity information (e.g., national ID) when register. House086 also has a medical board which oversees the clinical information and request submitted by the members.
